# Weight Loss Medication Marketing and First Nations People: Disease Awareness or Corporate Profit?

**DOI:** 10.5694/mja2.70246

**Published:** 2026-07-10

**Authors:** Troy Walker, Simone Sherriff, Jennifer Browne

**Affiliations:** ^1^ Institute for Health Transformation, Global Centre for Preventive Health and Nutrition, School of Health and Social Development, Faculty of Health Deakin University Geelong Victoria Australia; ^2^ Poche Centre for Indigenous Health, Faculty of Medicine and Health, University of Sydney Sydney New South Wales Australia

**Keywords:** advertising as topic, pharmaceuticals, public health education, social media

## Abstract

The growing popularity of glucagon‐like peptide‐1 receptor agonists (GLP‐1 RAs) has driven off‐label prescriptions and supply shortages, raising equity concerns for First Nations peoples disproportionately affected by diabetes. Pharmaceutical companies have simultaneously accelerated their marketing through disease‐awareness campaigns, sponsored events and telehealth models, with many campaigns prominently featuring women of colour, including First Nations women. In this perspective article, we argue, from a commercial determinants of health perspective, that pharmaceutical companies often influence perceptions of body image, medicines policy and prescribing without addressing the systemic conditions driving First Nations health inequity. We urge clinicians and policymakers to ensure decisions remain free from commercial influence.

## 1 Introduction

The commercial determinants of health, defined as the systems, practices and pathways through which commercial actors influence health and equity, are increasingly recognised as shaping population health outcomes, including for First Nations people [1, 2]. The pharmaceutical industry plays a central role in the discovery, development and manufacture of life‐saving medicines; however, harmful pharmaceutical industry practices, including inappropriate marketing, are well‐documented in the commercial determinants of health literature [3]. In Australia, the growing popularity of glucagon‐like peptide‐1 receptor agonist (GLP‐1 RA) medications for weight loss, alongside the government's recent commitment to list semaglutide on the Pharmaceutical Benefits Scheme (PBS) for eligible patients, has intensified public and media attention around these drugs and the companies that manufacture them [4]. The weight‐loss industry has a long history of targeting women through its marketing, and emerging promotion of GLP‐1 RA medications appears to follow similar patterns [5, 6]. Expanding medical weight‐loss promotion in Australia across multiple platforms, including on social media, podcasts and streaming services, raises concerns that such marketing may disproportionately affect women of colour, including First Nations women [6]. This perspective article was written by two First Nations researchers and one non‐Indigenous researcher (Box 1) as a part of a larger project (unpublished data) that investigates the influence of the pharmaceutical industry on First Nations peoples' health and well‐being and contemporary GLP‐1 RA marketing through the lens of the commercial determinants of health [1]. This larger project is reported elsewhere, following the CONSIDER statement (Supporting Information) [7].

BOX 1 **Researcher positionality statement.**

**Table:** 

Troy Walker is a Yorta Yorta man, clinician and researcher working in Aboriginal and Torres Strait Islander health for 16 years. Troy's cultural identity, clinical and lived experiences shape translational approaches to research with Community, emphasising strength‐based care and shared decision‐making. I acknowledge my responsibilities to Country, Community and Elders, and I remain committed to ensuring that our research benefits Aboriginal and Torres Strait Islander peoples
Simone Sherriff is a Wotjobaluk woman living on unceded Wiradjuri lands, and a mother of two, a research fellow at the University of Sydney, and has worked with the Aboriginal community‐controlled health sector for 13 years
Jennifer Browne is a non‐Indigenous researcher with a background in nutrition and public health. Her research is grounded in more than two decades of practice and partnership with the Aboriginal Community Controlled Health sector. She is committed to research practice that builds Aboriginal and Torres Strait Islander research leadership, while acknowledging her responsibility to remain accountable to the communities whose priorities her research is designed to serve

The need for actionable responses must include grounded co‐creation with First Nations community—moving beyond consultation and towards genuine shared governance, where priorities, messaging and systems are defined by community knowledge, values and lived experiences. Aboriginal Community Controlled Health Organisations (ACCHOs) must be involved as leaders in designing culturally safe approaches to obesity, diabetes and medicine use.

## 2 A Brief History of GLP‐1 RAs in Australia

First developed as diabetes medications, GLP‐1 RAs stimulate insulin secretion, slow gastric emptying, encourage appetite suppression and promote weight loss [8]. Exenatide was the first GLP‐1 RA medication approved in Australia, in 2007, and subsequently listed on the PBS for the treatment of type 2 diabetes [9]. Several other agents followed, including liraglutide, dulaglutide and semaglutide, which were PBS‐listed for type 2 diabetes in 2016, 2018 and 2020, respectively [10]. First Nations people are disproportionately affected by type 2 diabetes, with prevalence reported to be as high as 40% in some regions of Australia [11]. Yet evidence from ACCHOs and mainstream general practice suggests uptake of GLP‐1 RA in diabetes care for First Nations people has been low, despite their effectiveness in lowering glycated haemoglobin (HbA_1c_) levels and body weight [12]. At the time of these studies, these drugs were marketed for glycaemic control rather than weight loss. The 2021 approval of semaglutide for weight management in the United States, accompanied by widespread online and social media promotion, drove a surge in off‐label prescribing for weight loss [13]. This rapid increase in demand for semaglutide led to global supply shortages, including in Australia, reducing access for people with type 2 diabetes and raising concerns about equity for First Nations people [14].

In 2025, the Pharmaceutical Benefits Advisory Committee (PBAC) recommended PBS listing of semaglutide for patients with obesity and established cardiovascular disease [15]. Given that the cost of semaglutide treatment is about $4000 per year at private prescription prices, a PBS subsidy has the potential to improve equity of access for First Nations communities, who are disproportionately affected by obesity and cardiovascular disease yet often less able to afford the substantial out‐of‐pocket cost associated with these medicines [10]. Economic modelling suggests that increasing access to semaglutide for First Nations people with cardiovascular disease and obesity is likely to be cost‐effective and contribute to a reduction in cardiovascular events [16]. Notably, this analysis defined obesity as body mass index (BMI) > 27 kg/m^2^, whereas the PBAC recommended subsidising semaglutide for First Nations people with a BMI ≥ 32.5 kg/m^2^. Neither BMI threshold aligns with standard Australian classifications of obesity or severe obesity, as commonly reported in media coverage following the PBAC decision (Box 2) [17]. These recent developments illustrate two parallel and evolving processes influencing First Nations health in the past decade. On the one hand, PBS‐subsidised semaglutide may enhance equitable access to an effective treatment for diabetes, obesity and cardiovascular disease. On the other hand, expanding public subsidy of high‐cost medicines raises questions about sustainability in the context of finite health budgets and ongoing funding needs in preventive health, ACCHOs and community‐based programs. At the same time, reports of adverse effects and concerns about long‐term safety continue to emerge [18]. Furthermore, the rapid expansion of marketing and promotion of GLP‐1 RA products across Australian and international media platforms raises important ethical and public health questions about how, and to whom, these medicines are marketed.

BOX 2 **Australian Body Mass Index (BMI) Classifications.**

**Table:** 

BMI Classification (kg/m^2^) Underweight: < 18.5 Healthy weight: 18.5–24.9 Overweight: 25.0–29.9 Obese class I: 30.0–34.9 Obese class II: 35.0–39.9 Obese class III (severe obesity): > 40.0

## 3 The Power and Potency of Pharmaceutical Marketing

The surging popularity of GLP‐1 RAs has been accompanied by substantial growth in the revenues and market influence for the companies that produce them. It has been projected that global GLP‐1 RA sales will exceed US$100 billion by 2030 [19]. The market is dominated by Danish pharmaceutical company, Novo Nordisk, and United States‐based Eli Lilly. In June 2025, Novo Nordisk was reported to be the most valuable company in Europe by market capitalisation [20]. This scale of commercial growth facilitates substantial investment in promotional strategies. Previous analyses have demonstrated that many of the world's largest pharmaceutical companies allocate significant resources to sales and promotion, with some spending more on marketing than on research and development [21].

Some high‐profile GLP‐1 RA promotional campaigns and brand partnerships prominently feature women of colour. Recent examples include Oprah Winfrey's 2025 Australian tour, sponsored by Eli Lilly (https://www.oprahdaily.com/life/a65577109/oprah‐returns‐to‐australia‐this‐december/), and tennis champion, Serena Williams, appearing in the marketing campaign for telehealth company, Ro, which offers GLP‐1 RA treatment programs (https://ro.co/weight‐loss/serena/). In February 2026, Ro premiered a Superbowl advertisement that featured Serena Williams promoting her GLP‐1‐assisted weight loss during one of the most widely viewed sporting broadcasts of the year (https://ro.co/press/super‐bowl‐2026/). Although research from the United States indicates that pharmaceutical marketing can be racially patterned, the sociodemographic and regulatory context in Australia differs substantially [22, 23].

In Australia, the *Therapeutic Goods Act 1989* prohibits direct‐to‐consumer advertising of prescription medicines, including on social media [24]. However, pharmaceutical companies may lawfully promote prescription medicines to health professionals, engage with health care and patient organisations and undertake disease awareness campaigns, creating alternative channels of commercial influence [5, 25]. Concerns have been raised internationally, including in Australia, about the transparency of pharmaceutical industry payments to and engagement with clinicians and health consumer organisations [26]. Novo Nordisk Australia is engaging with First Nations people, organisations and consultancy firms, as evidenced through its Reconciliation Action Plan (RAP) and establishment of an Aboriginal and Torres Strait Islander Advisory Council [27]. Although leadership and governance from First Nations health professionals and academics may support culturally appropriate engagement, our research suggests that many First Nations people express reservations about RAPs and the commercial motivations of the pharmaceutical industry [28].

In the context of Australia's restriction of direct‐to‐consumer advertising, disease awareness campaigns function as an alternative promotional strategy for the pharmaceutical industry. Such campaigns enable GLP‐1 RA manufacturers to raise public awareness about obesity and encourage individuals to consult their doctor about medical weight loss without naming a specific medication. Although legally permissible in Australia, research suggests disease awareness campaigns may contribute to the medicalisation of body size and provide unbalanced information that generates consumer demand for unnecessary pharmaceutical treatment [5, 25]. Both Eli Lilly and Novo Nordisk are running disease awareness campaigns on Australian television, radio and social media, some of which feature women of colour (https://youtu.be/zemVcmTQFGw?si=ZCvkPdi5‐fyWRRKK, https://pro.novonordisk.com.au/wegovy‐landing‐page.html?referrer=https://www.bing.com/). The female authors of this article observed a striking volume of such marketing posts in their social media feeds over the summer holiday period, when health‐related resolutions are common. Further research is needed to determine whether First Nations women are being algorithmically targeted by this marketing.

At the same time, Australia has seen the expansion of direct‐to‐consumer telehealth platforms. Similar to Ro in the United States, these platforms offer access to GLP‐1 RA prescriptions through online medical weight loss programs, reshaping how prescription medicines are accessed and marketed. These platforms are themselves marketed on social media, including through campaigns featuring public figures such as First Nations singer Casey Donovan (https://www.myjuniper.com/blog/casey‐donovan‐joins‐juniper). Prescribing weight loss medications via telehealth has raised safety concerns, particularly for people living with eating disorders, which may disproportionately affect First Nations peoples [29].

## 4 Conclusion

We respect the autonomy of First Nations women, women of colour and all individuals who choose to use GLP‐1 RAs to meet their health needs. Our concern is not with personal health choices but with the commercial choices shaping them. We call on medical professionals to safeguard person‐centred care and shared decision‐making, ensuring that their prescribing decisions remain free from commercial influence. As trusted advocates within the health system, doctors have a responsibility to support regulatory settings that prioritise patients over pharmaceutical profits. This includes protections against pharmaceutical corporations capitalising on the health inequities faced by First Nations peoples. Ultimately, both clinical decisions and health policy should be based on the best available evidence, not shaped by the marketing practices of multinational pharmaceutical companies.

## Author Contributions

**Troy Walker:** concept and design, drafting, revising and editing, final approval of manuscript. **Simone Sherriff:** concept and design, revising and editing, final approval of manuscript. **Jennifer Browne:** concept and design, revising and editing, final approval of manuscript.

## Funding

This work was supported by National Health and Medical Research Council (2035274).

## Disclosure

Not commissioned; externally peer‐reviewed.

## Conflicts of Interest

The authors declare no conflicts of interest.

## Supporting information


**Data S1:** CONSIDER statement.

## Data Availability

This article includes no original data.
