# Supplementary material for: Weight Loss Medication Marketing and First Nations People: Disease Awareness or Corporate Profit?
Source: Med J Aust. 2026 Jul 10;224(7):e70246. doi: 10.5694/mja2.70246 (PMC13353042; doi:10.5694/mja2.70246)
Supplement: Supplementary file 1 — Data S1: CONSIDER statement. [file MJA2-224-0-s001.pdf]

The Medical Journal of Australia

Supporting Information

Supplementary material

This appendix was part of the submitted manuscript and has been peer reviewed.
It is posted as supplied by the authors.

Appendix to: T. Walker (Yorta Yorta), S. Sherriff (Wotjobaluk) and J. Browne. Weight Loss Medication Marketing and First Nations People: Disease Awareness or Corporate Profit? *Medical Journal of Australia* 2026; doi: 10.5694/mja2.70246.

CONSIDER Statement

Governance
1. Partnership agreements exist with Deakin University, Menzies School of Health Research, VACCHO, NACCHO, QAIHC,
2. All review mechanisms for all works as a part of the larger ACHIEVE project under which this work sits goes through an Indigenous Data Governance Group with First Nations members from our partner peak health organisations and university research team
3. Our research partnerships agreements include specific clauses on protecting IP/CP
Prioritisation
4. Research priorities in this perspective piece were encouraged through a combination of our First Nations Community Controlled Organisation Circle group within the broader ACHIEVE project and by First Nations participants in a related project looking at the pharmacy and pharmaceutical industries.
Relationships
5. The larger study was approved by both our university ethics committee, Deakin University Human Research Ethics Committee and the Aboriginal Health and Medical Research Council Human Research Ethics Committee.
6. The research was conceptualized and guided by First Nations researchers and evolved through discussions and yarns with ACCO partners and community members.
7. The broader research team (Murnong Health Research Mob) currently has 15 total members and 11 First Nations researchers with expertise in First Nations health, public health, clinical health and the commercial determinants of health.
Methodologies
8. This was a perspective piece and not primary research so a methodological category was not applicable.
9. N/A
Participation
10. N/A
11. N/A
12. N/A
Capacity
13. This perspective was lead authored by an Aboriginal researcher (TW) as a particular area of interest in a growing and relevant field for First Nations people. The work has enabled the primary author to build their knowledge in this area and help share the knowledge amongst other First Nations researchers and community members as a part of continuing professional development and education.
14. Professional development was ongoing with this work and building from the team's past research outputs and related projects and events. The first and second authors collaborated and yarned regularly about the influences and impacts in this perspective piece.
Analysis and Interpretation
15. N/A
Dissemination
16. Research dissemination will be through honouring knowledge translation principles and sharing all information with our broader internal research team (the Murnong Mob), ACCO partners, community members through regular committee group meetings, utilise conference presentations and discuss at our annual end of year gathering inviting partners and First Nations and allyship stakeholders.

17. We aim to further contribute and build upon our existing partnering relationships with this work, strengthen knowledges in the First Nations CDoH space, share and translate findings via media releases in oral and written form including long-form podcasts, radio segments, online mediums such as The Conversation or Croakey, social media sharing and direct presentations and workshops to our ACCO partner’s and interested community members
